# Giant lizards occupied herbivorous mammalian ecospace during the Paleogene greenhouse in Southeast Asia

**DOI:** 10.1098/rspb.2013.0665

**Published:** 2013-07-22

**Authors:** Jason J. Head, Gregg F. Gunnell, Patricia A. Holroyd, J. Howard Hutchison, Russell L. Ciochon

**Affiliations:** 1 Department of Earth and Atmospheric Sciences and Nebraska State Museum of Natural History, University of Nebraska-Lincoln, Lincoln, NE 68588, USA; 2 Division of Fossil Primates, Duke Lemur Center, 1013 Broad Street, Durham, NC 27705, USA; 3 Museum of Paleontology, University of California, Berkeley, CA 94720, USA; 4 Department of Anthropology and Museum of Natural History, University of Iowa, Iowa City, IA 52242, USA

**Keywords:** Squamata, gigantism, herbivory, paleoclimate, Eocene

## Abstract

Mammals dominate modern terrestrial herbivore ecosystems, whereas extant herbivorous reptiles are limited in diversity and body size. The evolution of reptile herbivory and its relationship to mammalian diversification is poorly understood with respect to climate and the roles of predation pressure and competition for food resources. Here, we describe a giant fossil acrodontan lizard recovered with a diverse mammal assemblage from the late middle Eocene Pondaung Formation of Myanmar, which provides a historical test of factors controlling body size in herbivorous squamates. We infer a predominately herbivorous feeding ecology for the new acrodontan based on dental anatomy, phylogenetic relationships and body size. Ranking body masses for Pondaung Formation vertebrates indicates that the lizard occupied a size niche among the larger herbivores and was larger than most carnivorous mammals. Paleotemperature estimates of Pondaung Formation environments based on the body size of the new lizard are approximately 2–5°C higher than modern. These results indicate that competitive exclusion and predation by mammals did not restrict body size evolution in these herbivorous squamates, and elevated temperatures relative to modern climates during the Paleogene greenhouse may have resulted in the evolution of gigantism through elevated poikilothermic metabolic rates and in response to increases in floral productivity.

## 1. Introduction

Modern terrestrial herbivore ecosystems are dominated by mammal faunas that originated with the evolution of ungulate folivores during the middle Eocene [1]. Conversely, herbivory is comparatively rare among extant squamates [2]. Squamates do not efficiently metabolize plant matter compared with mammals [3], and digestion requires elevated body temperatures which are correlated to large body size [2,4–6] and restriction to tropical climates for most taxa [7].

The relative roles of physiology and ecological pressures from mammals as constraints on upper body size limits of herbivorous lizards are unknown, however. Direct and indirect interactions with ungulates and carnivorans are known to limit distribution and densities of carnivorous squamates [8–10] and the largest extant herbivorous reptiles only occur in insular, mammal-free habitats [3,11], suggesting competitive exclusion or predation pressure may limit maximum body sizes. Conversely, squamate body size can be affected by ambient temperature and food resources [3,12], and maximum body sizes of extant taxa may be limited by Holocene climatic maxima.

Fossil squamates generally demonstrate similar size and diversity patterns as extant herbivores during the Cenozoic, but the squamate fossil record is poorly sampled and generally restricted to the mid and high latitudes of North America and Europe [13–16]. The relative paucity and geographical restriction of the squamate fossil record confounds efforts to examine the historical relationship between body size and environment relative to faunal competition, climate and historical contingency in poikilothermic herbivores.

Here, we describe a giant acrodontan lizard from the rich, low-latitude vertebrate fossil record of the late middle Eocene Pondaung Formation of central Myanmar that includes a diversity of eutherian mammals, turtles, squamates and crocodylians recovered from siliciclastic sediments representing fluvial depositional environments [17–21]. Analysis of the new acrodontan's inferred diet and estimated body mass in the context of the co-occurring fauna and in comparison to modern vertebrate communities allows us test the relative influences of mammalian competition versus climate regime as a regulating mechanism of herbivorous reptile body size by examining herbivore community structure in past and present vertebrate ecosystems and by estimating minimum paleotemperatures necessary to support a giant poikilothermic herbivore based on the mass-specific metabolic relationship between body size and climate in living herbivorous lizards.

## 2. Systematic paleontology

Squamata Oppel 1811

Iguania Cuvier 1817

Acrodonta Cope 1864 *sensu* Estes *et al*. 1988

*Barbaturex morrisoni* gen. et sp. nov.

### (a) Etymology

*Barbatus* (L) ‘bearded’ + *rex*, ‘king’, referring to the presence of ventral ridges along the mandible and giant size of the taxon. Species nomen honors Jim Morrison, vocalist and lizard king.

### (b) Holotype

**Figure 1.**
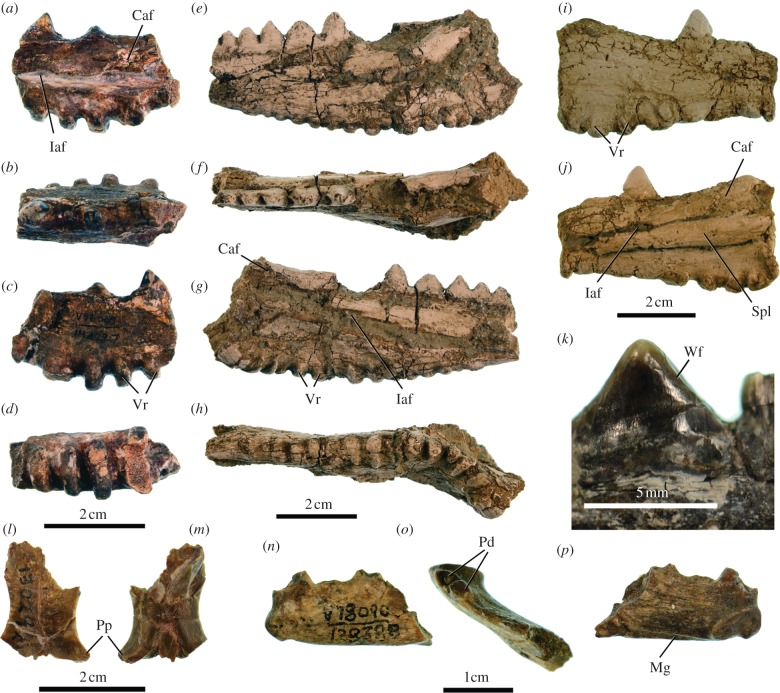
*Barbaturex morrisoni* gen. et sp. nov. (*a–d*) Holotype (UCMP 142227), right dentary in medial (*a*), dorsal (*b*), lateral (*c*) and ventral (*d*) views; (*e–h*) NMMP-KU 1923, left dentary (retrodeformed cast) in lateral (*e*), dorsal (*f*), medial (*g*) and ventral (*h*) views; (*i–j*) NMMP-KU 1925, right dentary (cast) in lateral (*i*) and medial (*j*) views; (*k*) UCMP 130290, posterior dentary tooth, in labial view; (*l–m*) UCMP 130292, parietal, in (*l*) dorsal, ventral (*m*) views; (*n–p*) UCMP 170491, left anterior dentary in lateral (*n*), dorsal (*o*) and medial (*p*) views. Abbreviations: Caf, articular facet for coronoid; Iaf, inferior alveolar foramen; Mg, Meckelian groove; Pd, pleurodont dentition; Pp, parietal process of frontal; Spl, splenial; Vr, ventral ridges; Wf, wear facets.

UCMP 142227 (University of California Museum of Paleontology), partial right dentary (figure 1*a–d*).

### (c) Referred specimens

UCMP 128388, anterior dentary; UCMP 128410, 130290, 130291, partial left dentaries; UCMP 130292, fused frontals assigned to the taxon on the basis of size; NMMP-KU 0092, partial left dentary; NMMP-KU 1923, partial left dentary; NMMP-KU 1924–1926, partial right dentaries [20] (figure 1*e–p*).

### (d) Locality and horizon

The type locality is UCMP V96009, a locality number used to designate a stratigraphically low purple mudstone underlying red beds at Thandaung kyitchaung [18,22], Pondaung Formation, northwest of Mogaung village, Sagaing District, Myanmar. Fossil-bearing beds of the Pondaung Formation near the village of Bahin have been dated to 37.2 ± 1.3 Ma. [23]. Referred specimen localities are UCMP V96009, V78090, PGN1, Kdw-42 (Kyawdaw area, [21]), Mgg-53A, B (Mogaung area, [21]), Tmk-35, Pondaung Formation, Sagaing District, Myanmar.

### (e) Diagnosis and description

Large bodied acrodontan lizard (approx. 100 cm snout-vent length = SVL) with a mandibular dental formula of two anterior pleurodont teeth and more than 10 mid- and posterior acrodont teeth. Posterior teeth are triangular with continuous wear facets, and lack accessory cusps (figure 1*e,k*). Wide, tall, anteromedially oriented ridges are present on the ventral margin of the anterior mandible (figure 1*a–j*), the dentary possesses a deep ventral extension below the Meckelian groove (figure 1*a,g,j*), the angular is fused to the dentary (see the electronic supplementary material, figure S1), the inferior alveolar foramen is formed by the dentary dorsally and splenial ventrally (figure 1*j*), the posterior mylohyoid foramen is absent, the Mecklian groove passes ventrally beneath the posterior margin of the mandibular symphysis, the anterior margin of the coronoid articular facet is just posterior to last tooth position (figure 1*a,g,j*), the parietal processes of the frontal are reduced and contribute less than 50 per cent of the posterior orbital margin (figure 1*l,m*). Additional descriptions are provided in the electronic supplementary material.

## 3. Material and methods

### (a) Phylogenetic analysis

**Figure 2.**
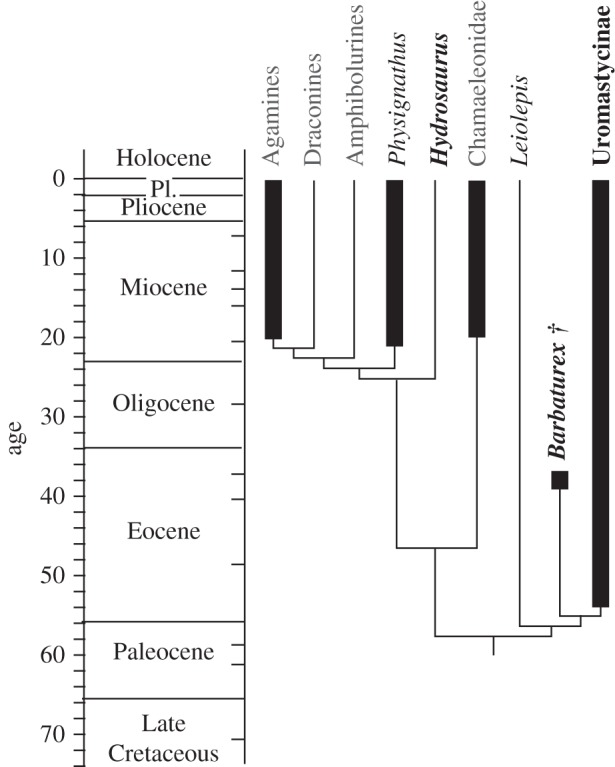
Temporally calibrated phylogenetic interrelationships of *Barbaturex morrisoni* relative to extant agamids based on morphological and molecular data [2,27]. Interrelationships of Chamaeleonidae is based on [25]. Thick vertical lines indicate known stratigraphic ranges. Name shades for extant taxa indicate feeding ecology: grey, predominately insectivory/carnivory; black, omnivory; bold, herbivory. First stratigraphic occurrence for agamines is from [28], *Physignathus* from [29], Chamaeleonidae from [30] and Uromastycinae from [31]. Divergence timing for the agamid total clade is from [32], *Leiolepis* is from [24].

Molecular and morphological data provide disparate hypotheses of the interrelationships of acrodontans, which limit the ability to resolve the phylogenetic status of fossil taxa [24]. To determine the interrelationships of *Barbaturex* to extant acrodontans *sensu* [25], we coded all preserved characters for the only extensive morphological phylogenetic analysis of constituent taxa [26]. Because *Barbaturex* remains preserve only a small fraction of described characters (5/122), we estimated the phylogenetic position of the taxon by optimizing character distributions onto tree topologies derived from combined morphological and molecular sequence data [2,27] (figure 2) and only molecular sequence data [33,34] (see the electronic supplementary material, figure S2). Phylogenetic position of *Barbaturex* was estimated by optimizing character distributions using Mesquite v. 2.75 [35]. Character codings for *Barbaturex* based on the matrix of [26] are as follows: 57(1), 58(0), 59(0), 65(2), 66(0).

### (b) Body size estimation

We estimated maximum body size measured as SVL in mm for *Barbaturex* by reduced major axis linear regression of natural log transformed measurements of SVL onto natural log transformed dentary lengths measured in a straight line from the anterior tip of the element to the posterior margin of the lateral coronoid process in mm for extant acrodontans (see the electronic supplementary material, table S1) using PAST v. 2.16 [36]. The resultant equation (LN SVL = 1.115*LN dentary length + 1.34, *R*^2^ = 0.89) was used to estimate body mass based on the general lizard SVL-mass equation of BM = 0.031*SVL^2.98^ [4].

### (c) Body mass comparisons

To examine the status of *Barbaturex* within the Pondaung vertebrate fauna, we ranked body masses of terrestrial herbivores, omnivores and carnivores and compared them to ranked masses for extant faunas that include the largest extant herbivorous squamates. Body masses were obtained from literature references (see the electronic supplementary material, tables S2–S5). Taxonomic and geographical range data for all extant mammals for comparisons with living herbivorous squamates is from [37], and body masses are from [38]. Maximum body mass reports and estimates for the largest extant herbivorous squamates, as well as Pondaung Formation mammalian body masses, are listed in the electronic supplementary material. Island endemic squamates [39,40] from faunas lacking folivorous mammals could not be compared with the Pondaung fauna, and completely arboreal, nocturnal and granivorous mammal taxa were not included in this analysis as their ecologies are not directly comparable to the examined squamates.

### (d) Paleotemperature estimation

Body size scales predictably with environmental temperature for a given mass-specific metabolic rate in poikilotherms [41,42]. We derived paleotemperatures as minimum mean annual temperature (MAT) for the Pondaung Formation from body size estimates of *Barbaturex* based on the relationship between SVL and minimum MAT for the largest living herbivorous squamates (see the electronic supplementary material, table S6) using the metabolic scaling equation from [42]:
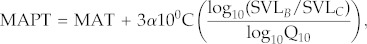
where MAPT is mean annual paleotemperature, SVL*_B_* is SVL for *Barbaturex*, SVL*_C_* is SVL for *Cyclura nubila*, MAT is minimum mean annual temperature within the geographical range of *C. nubila* (24.6°C), *α* is the metabolic scaling exponent of 0.33 [41], and Q_10_ is a mass-specific metabolic rate of 2–3 for reptiles [43]. We use *C. nubila* because it is the largest extant herbivorous lizard [5,7] and scaling the size-temperature model on it best fits the size-temperature distributions for other herbivorous taxa (figure 4).

## 4. Results and discussion

We assign *Barbaturex* to crown Acrodonta relative to priscagamines and more fragmentary stem taxa based on the character combination of reduced numbers of pleurodont anterior teeth, acrodont cheek dentition with deep, continuous interdental grooves and reduction of the splenial to the posterior region of the dentary [25,26,44–46]. Character optimization results in monophyly of *Barbaturex* + Uromastycinae for both morphological and molecular topologies, with tree lengths one to two steps shorter than all other alternate topologies based on the morphological + molecular scaffold (figure 2) and two to four steps shorter on the molecular scaffold (see the electronic supplementary material, figure S2). Character support for this hypothesis includes the absence of the posterior mylohyoid foramen and the ventral orientation of the Meckelian groove at the anterior tip of the dentary (figure 1*p*). The presence of two pleurodont dentary teeth [32] and a shortened parietal process of the frontal are additionally shared by the clade consisting of Uromastycinae, *Barbaturex*, and *Leiolepis* (figure 2).

The stratigraphic occurrence of *Barbaturex* is consistent with our hypothesis of interrelationships (figure 2). The oldest fossil records of unambiguous crown acrodontans consist of uromastycines from the early Eocene of Europe and Asia [31,47], *Barbaturex* in the late middle Eocene of Asia and possibly the lineage including extant *Leiolepis* from the late Eocene of North America [24]. The first occurrence of the clade including agamines, draconines and amphibolurines may be early middle Eocene [32], but the late middle Eocene age of the Pondaung record precedes the first occurrences of crown members of this clade, which are early Neogene in age, consistent with molecular divergence estimates [48].

We estimate a SVL of 981 mm ± 107 mm, and a mean body mass of 26.7 kg (range of 36.9–18.9 kg) for *Barbaturex* based on the relationship of dentary length to body size in extant taxa. *Barbaturex* was over twice as long as the largest extant agamid [7], and estimated body sizes are larger than all extant and known fossil terrestrial lizards with the exception of extant *Varanus komodoensis* [49] and extinct *V. priscus* and *Chianghsia nankangensis* [50–53].

**Figure 3.**
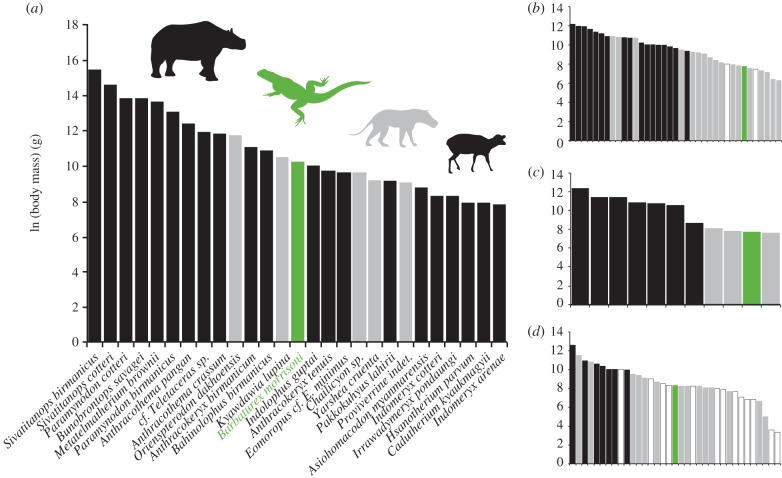
Ranked body masses of Pondaung Formation vertebrates compared with ranked masses of modern faunas that include the largest extant herbivorous squamates. (*a*) *Barbaturex morrisoni*, Pondaung Formation, Myanmar; (*b*) *Uromastyx aegypticus*, North Africa and Middle East; (*c*) *Hydrosaurus amboinensis*, Philippines; (*d*) *Ctenosaura similis*, Central America. Colours are: black, ungulates; grey, carnivorous mammals; green, squamates; white, insectivores and non-ungulate herbivores.

Body size, dental morphology and phylogenetic relationships of *Barbaturex* allow for inference of feeding ecology. Large body size is correlated to herbivory in squamates [5,54], except for the largest varanids, which are carnivores [10,55,56]. *Barbaturex* lacks dental adaptations for carnivory including recurved, serrated and laterally compressed teeth [57]. Instead, it possesses an acrodont dentition with precise shearing occlusion as indicated by continuous wear facets on mandibular dentition (figure 1). The same occlusal mechanism facilitates herbivory in extant agamids [58,59], and tooth crown morphology in *Barbaturex* is most similar to herbivorous adult *Hydrosaurus*. Herbivorous agamids will consume animal protein [60], and omnivorous agamids consume both plants and insects [61]. It is probable that *Barbaturex* would have opportunistically preyed on invertebrates; however, prey size scales with body size in carnivorous lizards, including iguanians [62] and large body size in *Barbaturex* probably precluded insectivory as a major component of feeding ecology, at least in mature individuals. The phylogenetic interrelationships of *Barbaturex* relative to crown agamids are also consistent with herbivorous feeding habits. Optimization of feeding habits on crown acrodontan phylogeny demonstrates that *Barbaturex* is nested within an omnivorous to herbivorous clade as the sister taxon to fully herbivorous Uromastycinae and bracketed by omnivorous *Leiolepis* (see figure 3 and electronic supplementary material, figure S2).

Body mass of *Barbaturex* falls approximately in the middle of size ranges for the Pondaung ungulate guild (figure 3*a*), and is larger than estimated body masses [63] for the smaller perissodactyls and most artiodactyls. The position of *Barbaturex* within the body mass distribution of the Pondaung vertebrate fauna is unique relative to extant herbivorous squamates. In all three examined modern faunas, there is no overlap in body mass between ungulate herbivores and squamates. Instead, squamate body masses are far smaller and fall within a range of carnivorous mammals, omnivores and non-ruminant herbivores for all modern faunas, including those from open, xeric environments (figure 3*b*), and both dry and wet forested environments (figure 3*c,d*). Difference in body size of *Barbaturex* relative to extant lizards cannot be explained by differences in ungulate body mass because Pondaung ungulates are both larger and smaller than extant taxa. Instead, body mass overlap between *Barbaturex* and Pondaung Formation mammals indicates that direct competitive exclusion or predation pressure did not restrict body size of these herbivorous squamates, despite differences in metabolic rate and dietary efficiency between poikilothermic and homeothermic herbivores. Similarly, indirect depression of biomass and diversity in extant squamates by environmental modification of ungulates does not appear to have been present in the Pondaung ecosystem based on both size and numbers of recovered specimens of *Barbaturex*.

**Figure 4.**
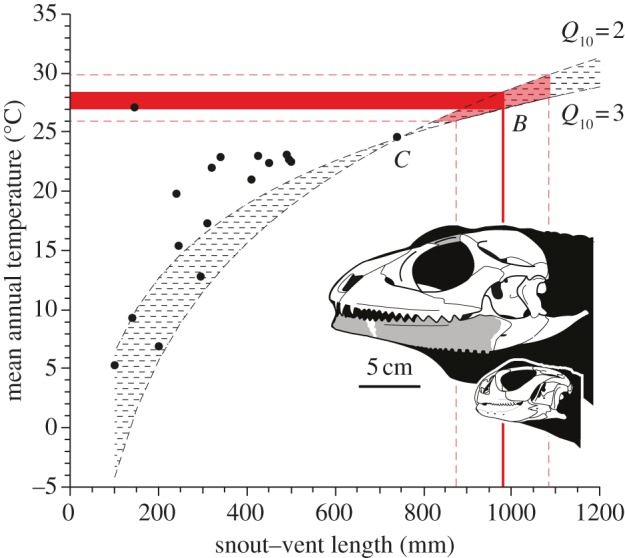
Minimum mean annual temperature (MAT) estimation of Pondaung Formation environments based on metabolic scaling of body size and environmental temperatures for modern herbivorous lizards and *Barbaturex morrisoni*. Model SVL-MAT scaling relationship (dashed areas) are based on maximum SVL and minimum MAT for *Cyclura nubila* (*C*) at *Q*_10_ values of 2 and 3. Solid red area represents temperature estimates for a snout vent length (SVL) of 981 mm for *Barbaturex morrisoni* (*B*). Dashed lines represent temperature values for SVL minima and maxima. Solid black dots represent SVL-MAT coordinate values for extant herbivorous lizards (see the electronic supplementary material, table S6). Inset, skull size of *Barbaturex morrisoni* compared with the largest extant agamid, *Uromastyx aegypticus*. Shaded regions represent known elements. Missing skull morphology based in part on *Hydrosaurus amboinensis*, size is derived from a dentary length of 14.5 cm based on UCMP 128388, NMMP-KU 0092, NMMP-KU 1923 and NMMP-KU 1925.

Based on the relationship of maximum body size to minimum MAT in extant herbivorous squamates, *Barbaturex* at 981 mm SVL would require minimum MATs of 27.0–28.4°C (range = 26.0–29.9°C for SVL range of 874–1088 mm) to maintain efficient metabolism (figure 4). The late middle Eocene was an interval of cooling from the Middle Eocene climatic optimum [64], but included ice-free poles and extremely warm sea surface temperatures (SSTs) of 22.4–20.5°C at 65°S [65] during the temporal interval spanning the radiometric age estimates of the Pondaung Formation [23]. Model latitudinal SST gradients for the middle Eocene indicate higher SSTs by 6°C relative to modern at a paleolatitude of 13° N [65, figure 3], consistent with MAT differences of approximately 2–5°C for Myanmar in the region of locality UCMP V96009 between the late middle Eocene and modern [66].

Elevated middle Eocene MATs would have allowed for the evolution of large body sizes for a given mass-specific metabolic rate, as inferred for other giant early Paleogene squamates [42], and would have resulted in greater floral productivity than modern ecosystems at low latitudes [67]. Larger body sizes produce increased thermal inertia and may have resulted in elevated temperature-dependent metabolic processes, including digestive efficiency and nutrient uptake [68]. Increased plant productivity affects body size in extant herbivorous iguanians [69], and the comparatively wider range of Pondaung Formation ungulate body mass relative to modern faunas also suggests high primary productivity (figure 3).

Convergent gigantism in *Barbaturex* and other Cenozoic squamates [42,53] as components of diverse vertebrate ecosystems demonstrates a greater past ecological breadth and diversity than expected from surveying extant herpetofaunas. These discoveries indicate that hypotheses of competitive advantage in extant mammals due to elevated metabolic processes are probably artefacts of modern climate and should not be used as models for inferring historical patterns of diversification and dominance in non-analogue deep time climates.
